# Unveiling the Mysteries of a Composite Compound Odontoma: Insights From the Management of a Rare Entity

**DOI:** 10.7759/cureus.52785

**Published:** 2024-01-23

**Authors:** Aakash Gupta, Tarun Kumar Singh, Ashish J Johnson, Rukhsar Showkat

**Affiliations:** 1 Conservative Dentistry and Endodontics, All India Institute of Medical Sciences, Bathinda, Bathinda, IND; 2 Dentistry, All India Institute of Medical Sciences, Bathinda, Bathinda, IND

**Keywords:** cystic enucleation, compound odontomas, hamartomatous malformation, hard structures, oral cavity

## Abstract

Odontomas are one of the slow-growing odontogenic tumors. They are not a true neoplasm and are considered to be hamartoma. Odontomas consist of four distinct tissues, i.e., enamel, dentin, pulp, and cementum. Odontomas develop from fibroepithelial and undifferentiated mesenchymal cells which are essential for the development of the tooth. These are mostly asymptomatic and are incidentally detected on routine radiographic examination. This case report presents a unique case of a composite compound odontoma in an adult patient with flaring of teeth. A 28-year-old male patient reported to the Department of Dentistry for the correction of spacing in the upper front tooth region. Prompt diagnosis and management, including odontoma removal and aesthetic correction, were initiated. This case highlights the possibility of the presence of malformed tooth-like structures associated with flaring of teeth. It also focuses on the need for increased vigilance in individuals undergoing aesthetic correction procedures in the anterior maxillary region.

## Introduction

The oral cavity is the doorway for diagnosing multiple diseases affecting the human body. Various tooth development anomalies can affect human dentition, with compound odontomas being one of them. Odontomas are the most common odontogenic tumors after ameloblastoma, accounting for about 20% of all cases [1]. However, statistics might change as most odontomas go unnoticed. In most cases, it is found impacted within the jaw, but it may erupt into the oral cavity. Sometimes, it can be misdiagnosed as a supernumerary tooth. Odontomas have been observed in cases with trauma during deciduous dentition along with inflammatory processes and congenital abnormalities (Gardner syndrome, Hermann syndrome) and variations in the genetic factors responsible for regulating tooth development [2]. Prevalence is more common in females with a higher probability toward the anterior maxilla [3]. Two forms of odontomas have been commonly reported, namely, compound and complex. Compound odontomas on radiographs simulate small tooth-like entities, whereas complex odontomas represent areas of radiopaque masses with varying densities. These malformations are benign and no recurrence has been reported yet [4]. In this case report, we present the case of a compound odontoma in the anterior maxilla of an adult patient.

## Case presentation

**Figure 1:**
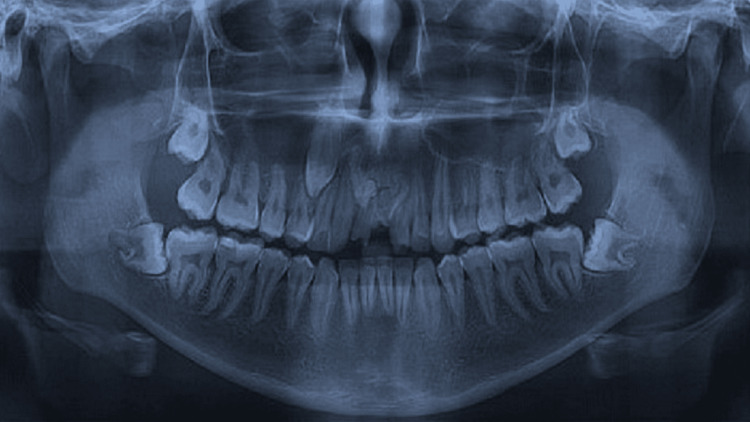
Orthopantomogram showing tooth-like structures in the anterior maxillary region.

A 28-year-old male patient reported to the Department of Dentistry with the chief complaint of spacing and flaring of his upper front teeth. The patient did not experience any pain or sensitivity to hot and cold in the concerned region. On panoramic radiographic evaluation (NewTom GO2D with NNT software), tooth-like entities (Figure 1) with thin radiolucent rims were observed apically to both central incisors in the anterior maxilla. All third molars were impacted along with the right maxillary canine.

**Figure 2:**
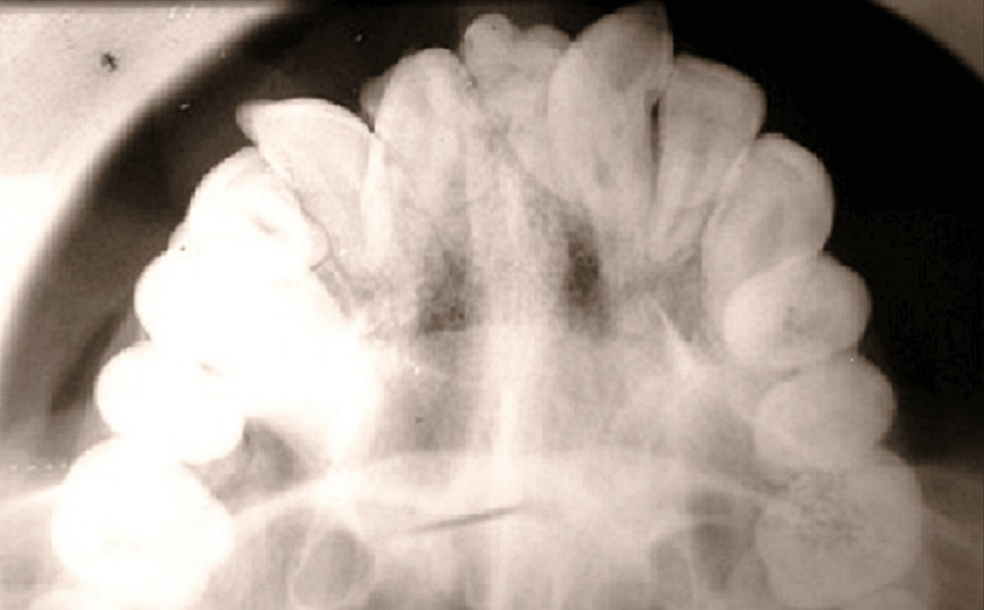
Occlusal radiograph showing tooth anomalies.

An occlusal radiograph (Figure 2) was planned to determine the anteroposterior location of the odontomas in the jaw.

**Figure 3:**
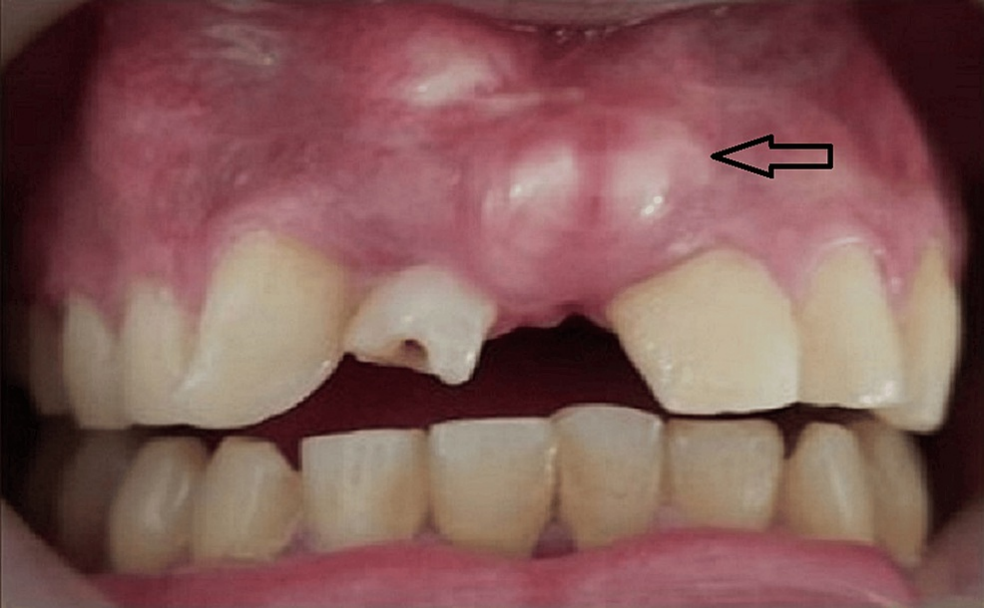
Hard tissue protuberances seen clinically, as shown by the arrow.

On intraoral examination, some hard tissue protuberances were felt on the buccal mucosa in the anterior maxilla region (Figure 3).

Surgical enucleation of these odontomas was planned followed by aesthetic rehabilitation. The patient was informed about the procedure and a consent form was signed. All necessary investigations were done before surgery and surgery was initiated.

**Figure 4:**
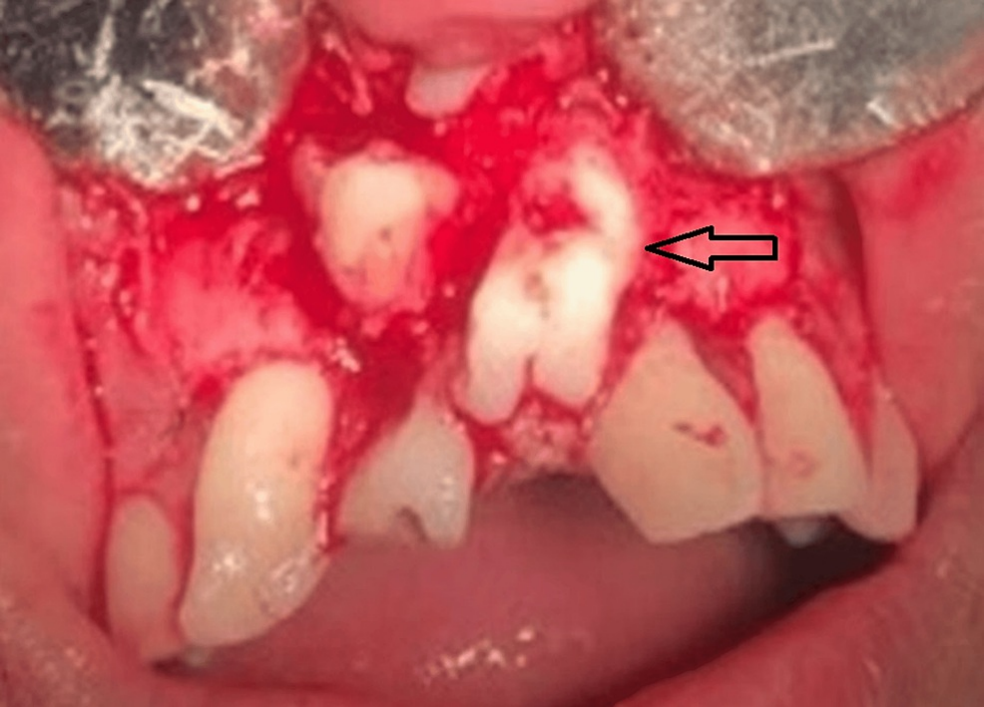
Odontomas seen after raising a mucoperiosteal flap, as shown by the arrow.

During surgery, 2% lignocaine with 1:200,000 adrenaline concentration was used for local anesthesia. After achieving profound anesthesia, two vertical incisions were made along with a gingival crevicular incision. A full-thickness mucoperiosteal flap was raised and odontomas were located (Figure 4).

**Figure 5:**
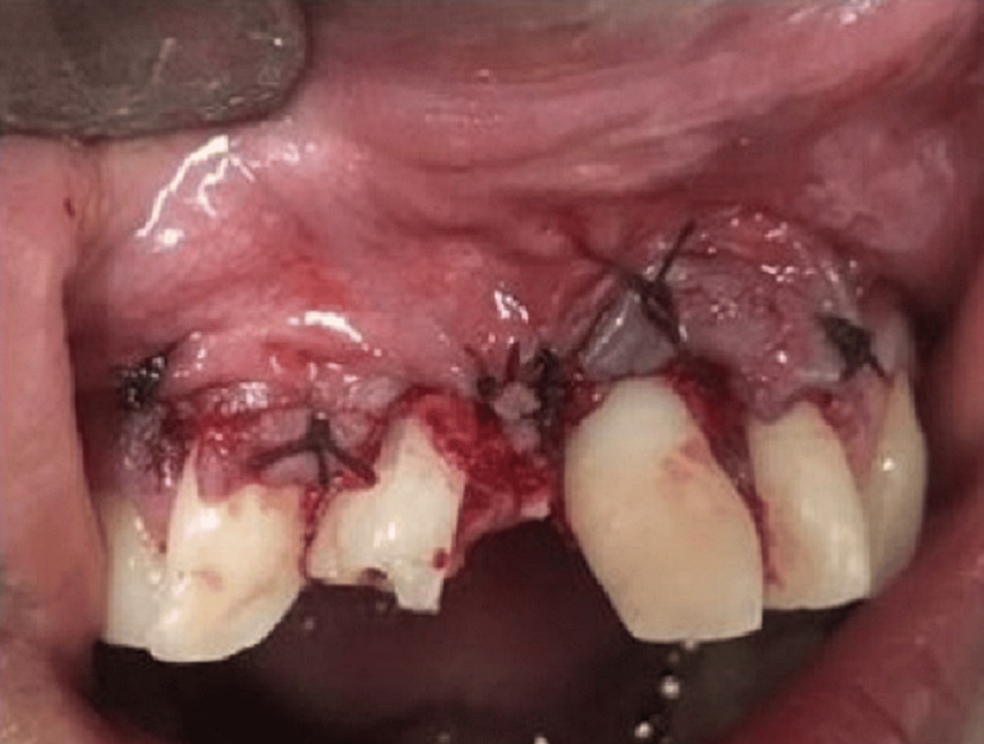
Sutures placed after odontoma removal.

Odontomas were then removed, and the flap was repositioned followed by suture placement (Figure 5).

**Figure 6:**
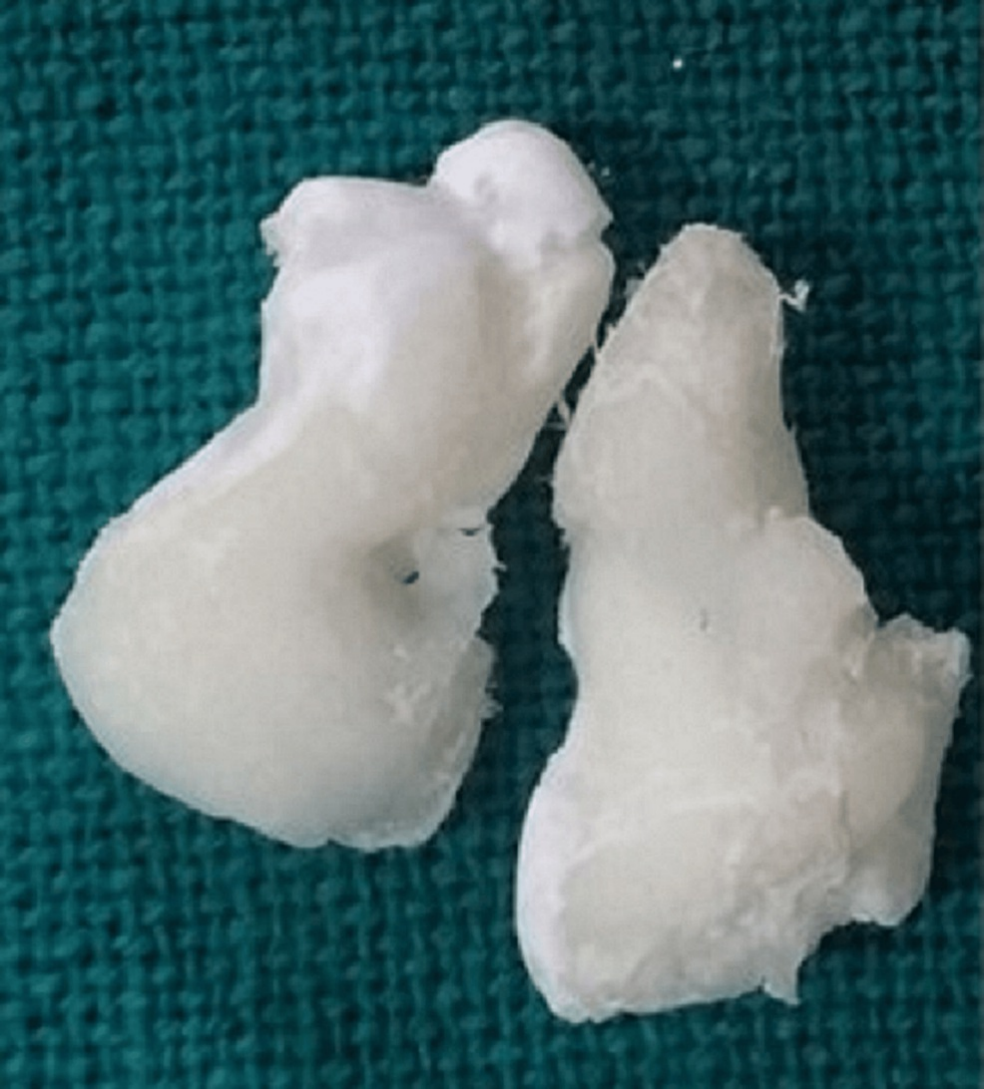
Odontomas seen clinically.

On visual examination, the odontomas resembled tooth-like entities (Figure 6).

**Figure 7:**
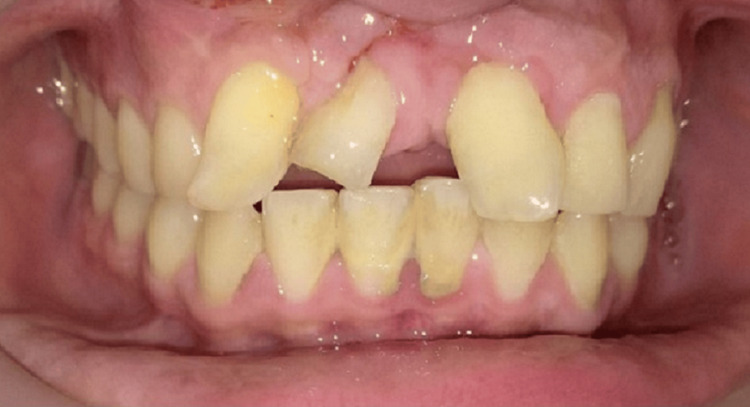
Postoperative healing after suture removal.

**Figure 8:**
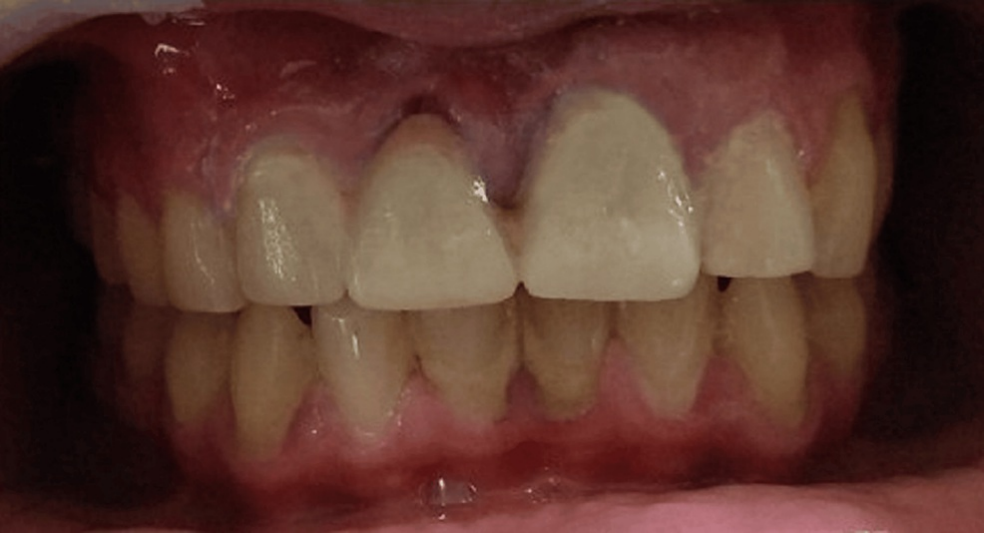
Aesthetic management of the anterior region.

On radiographical examination, it resembled components of a tooth. Systemic antibiotics and analgesics were advised to the patient along with postoperative instructions. The patient was recalled after 10 days to evaluate postoperative healing (Figure 7), followed by dental aesthetic management (Figure 8).

## Discussion

Odontomas are steady-growing, symptomless malformations found in jaws. In most instances, odontomas are found impacted in the jaws and rarely erupt into the oral cavity [5]. Their number can vary from a few to hundreds. They also vary in size from a few millimeters to centimeters, resulting in bone expansion. Mostly compound odontomas are smaller in size compared to complex odontomas [6]. Odontomas are benign as their growth becomes stagnant when they mature. There are various clinical aspects associated with odontoma, including intra‐bony, extra‐bony, and erupted types. In general, the majority are intraosseous. It is worth noting that the anterior region of the maxilla (67%) is the most commonly affected region.

Even though are asymptomatic, some clinical signs may depict the presence of odontomas such as retention of primary teeth, impaction of permanent teeth, cortical bone expansion, tooth displacement, flaring of teeth, and change of occlusion [7]. Although it is very common in the young population, it can be seen in adults.

Histologically, odontoma comprises odontogenic soft and hard tissues such as enamel, dentin, cementum, and pulpal tissue [8]. The compound odontoma denotes the identity of a normal tooth, while the complex odontoma presents as a disordered mass of hard odontogenic tissue. Loosely bound myxoid connective tissue with epithelial cell rests may be seen in proximity to these lesions [9]. The most accepted treatment is surgical enucleation of compound odontomas due to their encapsulation and if left untouched can lead to the formation of dentigerous cysts [10,11]. Early detection and effective treatment enable us to adopt a more cautious approach during the surgical procedure, preventing the deterioration of the affected area and preserving the health and positioning of the neighboring tooth. Ultimately, this guarantees a favorable prognosis for the patient.

In this particular case, flaring of teeth was the chief complaint, which can be a sign of the presence of odontoma in the anterior maxillary region. Hence, if a patient presents with this, proper evaluation needs to be done to determine the etiology and diagnose this condition.

## Conclusions

This case report highlights the presence of a rare case of compound odontoma in the anterior maxillary region in a patient with flaring of central incisors. Proper vigilance in such cases emphasizes the possibility of compound odontomas in patients who undergo dental aesthetic procedures, especially in the anterior maxilla. This case highlights the prompt diagnosis and interdisciplinary approach necessary to treat such cases.
